# Unlocking the Anticancer Potential of Patchouli Leaves: Molecular Mechanisms and Translational Perspectives

**DOI:** 10.3390/molecules31162870

**Published:** 2026-08-17

**Authors:** Elshan Musazade, Lizhu Qin, Fengshuo Yu, Nan Li, Liquan Guo, Chunyu Zhang

**Affiliations:** 1 College of Life Sciences, Jilin Agricultural University, Changchun 130118, China; elshan.musazade1@gmail.com (E.M.); m18978026670@163.com (L.Q.); 15947877288@163.com (F.Y.); 13504494295@163.com (N.L.); 2 Key Laboratory of Soybean Molecular Design Breeding, Northeast Institute of Geography and Agroecology, Chinese Academy of Sciences, Changchun 130102, China; 3 School of Food and Biology, Changchun Vocational University, Changchun 130033, China

**Keywords:** patchouli leaves, *Pogostemon cablin*, anticancer activity, molecular mechanisms, translational oncology

## Abstract

Cancer remains one of the leading causes of global mortality, with its incidence continuing to rise due to population growth, aging, lifestyle factors, and environmental exposures. Despite significant advances in early diagnosis and therapeutic strategies, the clinical management of cancer is still hindered by drug resistance, limited selectivity, and treatment-related toxicity. Consequently, increasing attention has been directed toward natural products as sources of novel anticancer agents with improved efficacy and reduced adverse effects. *Pogostemon cablin* (patchouli), a medicinal plant widely used in traditional medicine, has emerged as a promising candidate owing to its diverse bioactive constituents and broad pharmacological properties. This review systematically summarizes and critically evaluates current evidence on the anticancer potential of patchouli leaves, with particular emphasis on molecular mechanisms and translational relevance. Based on available experimental and preclinical studies, patchouli and its major phytochemicals exhibit notable anticancer activity against a wide range of malignancies, including endometrial, ovarian, liver, skin, nasopharyngeal, prostate, hematological, colorectal, and lung cancers. Mechanistically, these effects are primarily associated with the modulation of apoptosis, cell cycle regulation, oxidative stress, and key oncogenic signaling pathways, as well as potential synergistic interactions with conventional chemotherapeutic agents. Overall, this review highlights the therapeutic promise of patchouli leaves as a source of anticancer agents, identifies current knowledge gaps, and outlines future research directions to facilitate their development and clinical translation.

## 1. Introduction

Cancer remains one of the leading causes of morbidity and mortality worldwide, with an estimated 19.3 million new cases and nearly 10.0 million deaths reported in 2020. In Indonesia alone, approximately 396,941 new cancer cases and 234,131 cancer-related deaths were recorded during the same year [1]. Despite advances in cancer-specific treatment strategies, conventional therapies, including surgery, radiotherapy, chemotherapy, hormone therapy, and targeted therapy, remain the primary treatment modalities [2,3]. However, these approaches are frequently associated with serious adverse effects, including bone marrow suppression, gastrointestinal disorders, cardiotoxicity, hepatotoxicity, nephrotoxicity, and the development of therapeutic resistance, all of which limit treatment efficacy and compromise patients’ quality of life [2,4,5,6]. Consequently, there is an increasing demand for safer, more effective therapeutic agents that target multiple pathways involved in cancer progression.

Natural products have long served as an important source of anticancer drug discovery because of their structural diversity, broad biological activities, and relatively favorable safety profiles [7,8]. Approximately 40% of approved commercial drugs and 64.9% of currently available anticancer agents are derived from natural products or their derivatives [9,10]. Consequently, medicinal plants have attracted considerable attention as sources of bioactive compounds that modulate multiple molecular targets involved in tumor initiation, proliferation, metastasis, and apoptosis [11].

*Pogostemon cablin* (patchouli) is a medicinal plant widely cultivated in the Philippines, Malaysia, India, and China and has been used in traditional medicine for centuries. The dried aerial parts (Pogostemonis Herba) have traditionally been employed to treat fever, headache, nausea, diarrhea, gastrointestinal disorders, chronic weakness, and fatigue [12]. In addition to its traditional medicinal use, patchouli is widely used in pharmaceutical, cosmetic, and aromatherapy applications due to its antioxidant, anti-inflammatory, antimicrobial, and skin-protective properties [13,14,15].

Phytochemical investigations have revealed that patchouli leaves contain a diverse array of bioactive constituents, including terpenoids, flavonoids, lignans, phytosterols, pyrones, glycosides, aldehydes, alcohols, and organic acids [16,17,18]. Among these, patchouli essential oil (PEO) is the most extensively studied phytochemical fraction and contains several biologically active constituents that have attracted increasing research interest [19,20,21]. Emerging evidence indicates that patchouli extracts and their major phytochemicals possess antioxidant, anti-inflammatory, antiproliferative, pro-apoptotic, and other pharmacological properties relevant to cancer prevention and treatment, highlighting patchouli as a promising source of novel anticancer agents [12,22,23,24,25,26,27,28].

This review summarizes the current evidence regarding the anticancer potential of patchouli, with particular emphasis on its bioactive constituents and the molecular mechanisms underlying their anticancer activities. It further discusses the therapeutic potential of patchouli-derived compounds across different cancer types, identifies current knowledge gaps, and highlights future research directions to facilitate the development and clinical translation of patchouli-based anticancer therapies.

## 2. Materials and Methods

A comprehensive literature search was conducted using PubMed (U.S. National Library of Medicine, Bethesda, MD, USA; https://pubmed.ncbi.nlm.nih.gov/, accessed on 7 August 2026), Web of Science (Clarivate Analytics, Philadelphia, PA, USA; https://www.webofscience.com/wos/, accessed on 7 August 2026), Scopus (Elsevier B.V., Amsterdam, The Netherlands; https://www.scopus.com, accessed on 7 August 2026), Google Scholar (Google LLC, Mountain View, CA, USA; https://scholar.google.com/, accessed on 7 August 2026), and China National Knowledge Infrastructure (CNKI) (Tsinghua Tongfang Knowledge Network Technology Co., Ltd., Beijing, China; https://www.cnki.net, accessed on 7 August 2026) to retrieve peer-reviewed studies published up to June 2026 regarding the anticancer potential of *P. cablin*. Search terms included “*Pogostemon cablin*”, “patchouli”, “patchoulol”, “pogostone”, “pachypodol”, “cancer”, “anticancer”, “apoptosis”, “cell cycle”, “metastasis”, and “molecular mechanism”, alongside additional related keywords, which were logically combined using standard Boolean operators. Eligible publications comprised original research articles that investigated the anticancer effects of *P. cablin* extracts, essential oil, or isolated bioactive compounds, as well as relevant phytochemical and molecular mechanistic studies. Duplicate records, conference abstracts, editorial materials, and studies with insufficient experimental evidence were excluded. The remaining eligible literature was critically assessed and systematically synthesized to summarize current research progress and highlight prospective future research directions.

## 3. Taxonomic and Morphological Features of *Pogostemon Cablin*

*P. cablin* (Blanco) Benth., commonly known as patchouli, belongs to the family Lamiaceae, a major angiosperm lineage comprising 12 subfamilies, approximately 240 genera, and nearly 7200 recognized species [29,30]. The genus *Pogostemon* comprises around 80 species, primarily found in Southeast Asia [12,31].

Patchouli, a tropical dicotyledonous plant species, is believed to have originated in the Philippines. However, it is now widely cultivated throughout Southeast Asia, South America, and parts of West Africa [32,33]. The plant thrives in warm, humid conditions, with optimal growth at temperatures between 24 °C and 28 °C, a relative humidity of around 75%, annual rainfall of 2000–3000 mm, and altitudes from sea level to 1200 m [12,34].

Morphologically, *P. cablin* is a robust, bushy perennial herb well-adapted to warm, humid environments. Plants typically reach heights of 1.0–1.2 m and possess erect stems with elliptical, petiolate leaves measuring approximately 0.1 m in length and 0.02 m in width [12]. The leaves display shallow lobes with crenate-serrate margins and an obtuse apex [33]. Numerous epidermal trichomes, concentrated mainly on the abaxial leaf surface and along the veins, constitute the primary sites of PEO accumulation [12]. The plant develops a deeply penetrating, highly branched root system and produces small, pale pinkish-white flowers [35] while its leaves, flowers, and seeds exhibit a characteristic aromatic profile [36].

## 4. Phytochemical Composition of Patchouli Leaves

**Figure 1:**
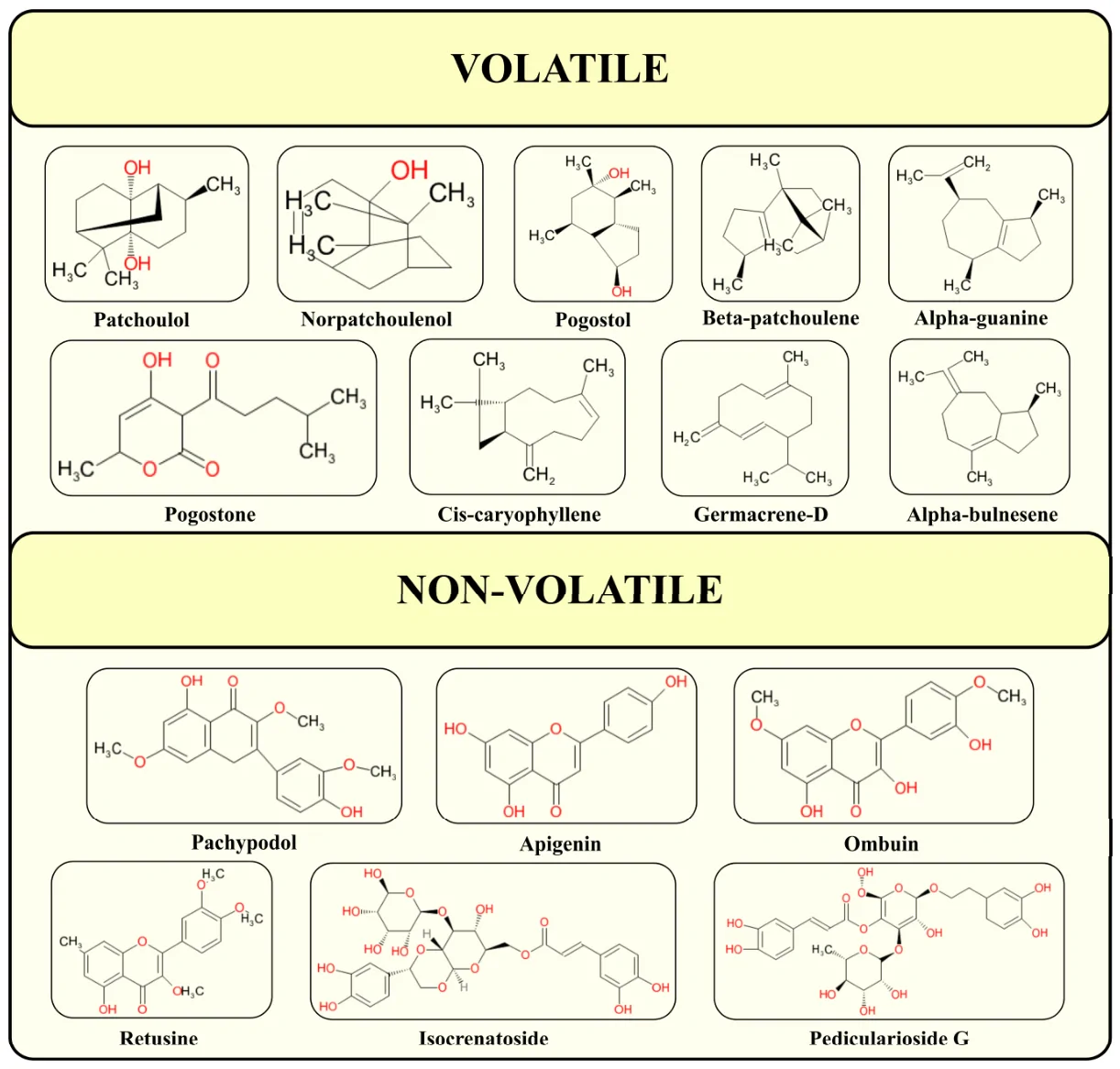
Chemical structures of representative volatile and non-volatile constituents in patchouli leaves. Volatile compounds are primarily sesquiterpenes and their oxygenated derivatives, whereas non-volatile compounds mainly comprise flavonoids and phenylethanoid glycosides.

Recent research has primarily focused on the patchouli plant as a whole, whereas detailed investigations of leaf-specific phytochemistry remain limited [12,37]. Nevertheless, patchouli leaves are recognized as chemically complex matrices that contain both volatile constituents (monoterpenes and sesquiterpenes) and non-volatile constituents (glycosides, flavonoids, organic acids, secondary metabolites, and phenolic compounds) (Figure 1) [35,38].

PEO represents the most extensively studied volatile fraction of the leaves and is well known for its broad pharmacological activities [39]. More than 150 compounds have been identified in PEO (Table 1) [40], with sesquiterpenes constituting the dominant class, alongside ketones, alcohols, and related derivatives [39,41]. Major constituents include patchoulene, patchoulol, pogostone, pogostol, bulnesene, caryophyllene, guaiene, and norpatchoulenol, among which patchoulene and patchoulol occur at the highest levels and serve as key indicators of oil quality, bioactivity, and commercial value [42,43]. The characteristic aroma and biological properties of PEO are primarily attributed to patchoulol, pogostone, patchoulene, and other sesquiterpene hydrocarbons such as guaiene and seychellene [37,44]. Additional volatile components, including germacrene-B, cardinene, daucosterol, and minor monoterpenes, have been identified through GC and GC-MS analyses, together with oxygenated sesquiterpenes such as β-caryophyllene, α-humulene, germacrene-D, elemene, patchoulene isomers, and caryophyllene oxide [45,46].

In addition to volatile constituents, patchouli leaves contain a diverse array of non-volatile compounds, with more than 50 components identified to date [57]. These include flavonoids, lignans, terpenoids, glycosides, organic acids, aldehydes, sterols, and triterpenoids (Table 2) [58,59]. Phytochemical analyses of the aerial parts, particularly the leaves, have revealed numerous flavones and related compounds, such as 4′,5-dihydroxy-7-methoxyflavone, 5,7-dihydroxy-3′,4’-dimethoxyflavone, 5-hydroxy-4′,7-dimethoxyflavone, 5-hydroxy-3′,4′,7-trimethoxyflavone, 4’,5,7-trihydroxyflavone, 5-hydroxy-3’,4’,7-trimethoxyflavanone, licochalcone A, 3,5-dihydroxy-4’,7-dimethoxyflavone, and ombuin [12]. Other notable non-volatile constituents include tilianin, stigmasterol, retusine, tschimganical A, dibutyl phthalate, and pachypodol, the latter receiving increasing attention due to its reported bioactivities [60,61].

Patchouli leaves contain prominent non-volatiles, including pedicularioside G, isocrenatoside, glycosides, verbascoside, actinosides, campeoside, and cablinoside epimers A and B [57,68]. Extensive solvent extraction combined with chromatographic and spectroscopic techniques has enabled the isolation of additional flavonoid glycosides, sterols, organic acids, and triterpenoids [69,70,71]. Advanced analytical approaches, including HPLC-Q-TOF-MS, HPLC, TLC, HSCCC, and preparative HPLC, have further facilitated the comprehensive characterization and quantification of these compounds [65,72,73]. Collectively, these findings underscore the exceptional chemical diversity of patchouli leaves and provide a robust phytochemical basis for elucidating their biological and anticancer potential.

## 5. Pharmacological Activities

Patchouli leaves exhibit a wide range of pharmacological effects attributable to their chemically diverse bioactive constituents. Substantial evidence supports their antimicrobial potential, including antibacterial, antifungal, and antiviral activities, with reported *in vitro* antiviral activity against pathogens such as the influenza virus and human immunodeficiency virus (HIV) [74,75,76]. In addition, these leaves demonstrate notable gastrointestinal protective effects, including antiemetic activity, regulation of intestinal function, and protection against peptic ulcers and diarrhea [77,78].

Documented cardiovascular and hematological activities include modulation of fibrinolysis and blood coagulation, as well as antihypertensive and antithrombotic effects [37,79]. Patchouli leaves also exhibit multiple protective and therapeutic properties, including antioxidant, analgesic, insecticidal, anti-inflammatory, and aphrodisiac effects, and have been used in dermatological treatments, aromatherapy, and as aphrodisiacs [80,81]. Emerging evidence further indicates roles in metabolic regulation, including anti-obesity effects, modulation of gut microecology, and antidiabetic activity, highlighting their systemic pharmacological significance [82,83].

Collectively, these findings suggest that patchouli leaves exert broad biological effects at cellular, tissue, and systemic levels. Consequently, growing attention has been directed to their potential roles in cancer prevention and therapy, warranting further investigation of their anticancer properties.

## 6. Anticancer Activity

**Figure 2:**
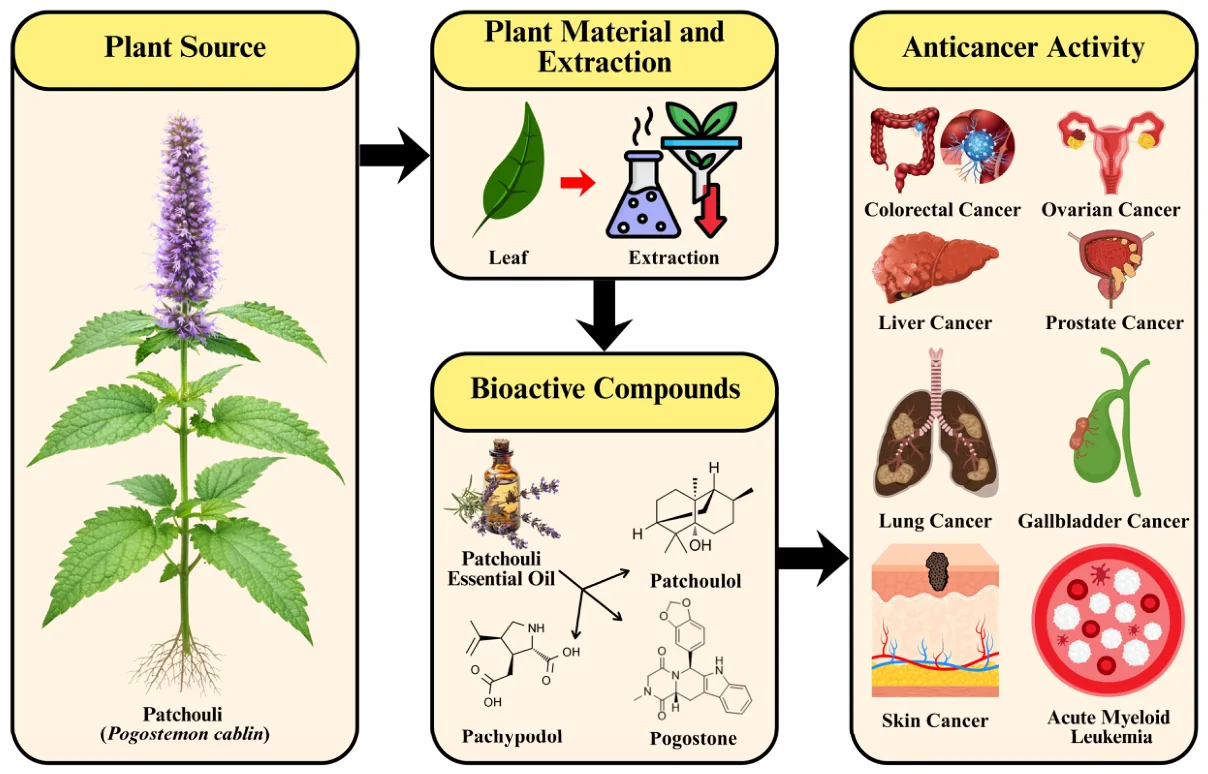
Overview of patchouli-derived bioactive compounds and their anticancer activities. This schematic illustration summarizes the source, extraction process, and anticancer potential of patchouli. The leaves of patchouli serve as the primary plant material for extraction, yielding bioactive constituents such as PEO and its major compounds, including patchoulol, pogostone, and pachypodol. These phytochemicals exhibit broad-spectrum anticancer activity against various malignancies, including colorectal, ovarian, liver, prostate, lung, gallbladder, and skin cancers, as well as acute myeloid leukemia, underscoring the therapeutic potential of patchouli-derived compounds as natural anticancer agents.

Emerging experimental evidence has highlighted patchouli leaves as a promising source of anticancer agents. Several bioactive compounds, such as patchoulol, pachypodol, and pogostone, have shown significant anticancer effects across various cancer types, including liver, colorectal, lung, prostate, and skin cancers. Their anticancer activity is primarily mediated through antiproliferative and apoptosis-inducing mechanisms, offering a mechanistic basis for the tumor-suppressive effects observed in preclinical models [72,84]. The reported anticancer effects of patchouli-derived bioactive compounds across different cancer models are systematically compiled in Table 3, while an overview of these bioactive compounds and their associated anticancer activities is summarized in Figure 2.

### 6.1. Anticancer Activity Against Endometrial Cancer

Endometrial cancer is the sixth most frequently diagnosed malignancy among women worldwide. It is the most prevalent gynecological cancer in developed countries. Due to increasing life expectancy, lifestyle changes, and associated risk factors, the global incidence of endometrial cancer has been steadily rising in recent years. In 2021 alone, approximately 97,370 new cases of endometrial cancer were reported worldwide [99].

Experimental studies have shown that *P. cablin* aqueous extract (PCAE) exhibits significant anticancer activity in endometrial cancer models, particularly in human Ishikawa endometrial cancer cells. The MTT assay was used to evaluate cell proliferation after PCAE treatment, while flow cytometry (FACSCalibur) was employed to analyze DNA content, cell-cycle distribution, and apoptosis-related changes. Furthermore, the expression and activation of apoptosis-associated proteins, including caspase-3 (CASP3), CASP9, and apoptosis-inducing factor (AIF), were assessed by Western blot analysis [90].

Treatment with PCAE (0, 1, 2, and 4 mg/mL) resulted in a dose-dependent suppression of Ishikawa cell proliferation and a marked increase in apoptosis. These effects were accompanied by increased CASP3 activity, CASP9 activation, and AIF involvement, collectively indicating the engagement of intrinsic apoptotic pathways. Further gene expression profiling (GEP) revealed that, in addition to suppressing endometrial cancer cell proliferation, patchouli-derived constituents exerted broader antitumor regulatory effects in established endometrial cancer cells [90].

These findings collectively demonstrate that PCAE demonstrates pronounced anticancer effects against endometrial cancer by inhibiting tumor cell proliferation and triggering intrinsic apoptosis. This is achieved through coordinated activation of CASP-dependent pathways and apoptosis-inducing factor-mediated signaling, underscoring its potential as a promising plant-derived therapeutic agent for the management of endometrial cancer.

### 6.2. Anticancer Activity Against Ovarian Cancer

Ovarian cancer is the third most prevalent malignancy and is the deadliest cancer affecting the female reproductive system. Around 70% of patients receive their diagnosis at advanced stages of the disease (FIGO stages III–IV), often with distant metastasis [100]. The elevated mortality associated with ovarian cancer is largely attributable to late-stage diagnosis and the absence of reliable early detection methods [101,102]. Even with the standard-of-care treatment, which includes optimal cytoreductive surgery followed by adjuvant chemotherapy, most patients eventually develop recurrent, chemotherapy-resistant disease, leading to a global 5-year survival rate of merely 30–40% [103].

Recent studies indicate that pogostone, a major bioactive constituent of patchouli, exerts potent anticancer effects in ovarian cancer models. Its activity was evaluated in OVCAR-3 human ovarian cancer cells treated with pogostone at the IC_50_ concentration of 90 µg/mL for 24 and 48 h. Apoptotic responses and cell viability were assessed using the MTT assay and flow cytometry, respectively. In contrast, PCR and a CASP3 colorimetric assay were employed to examine gene expression associated with apoptosis and cell-cycle regulation, as well as CASP3 protein levels [96].

Pogostone exposure resulted in a marked increase in apoptosis in OVCAR-3 cells at both time points. Molecular analyses revealed significant upregulation of tumor suppressor genes, including *PTEN* and *DACT1*, as well as apoptosis-related genes *CASP8*, *CASP9*, and *CASP3*. A significant elevation in the ratio of pro-apoptotic *BAX* to anti-apoptotic *BCL-2* was observed. In contrast, key cell-cycle regulators, such as *CCND1* and *CDK4*, were significantly downregulated. These transcriptional changes were supported by increased CASP3 protein expression, as confirmed by the colorimetric assay [96].

Collectively, these results demonstrate that pogostone effectively suppresses ovarian cancer cell proliferation and promotes apoptosis by coordinating the activation of tumor suppressor signaling, caspase-dependent apoptotic pathways, and the disruption of cell-cycle regulatory mechanisms, underscoring its promise as a plant-derived therapeutic candidate for ovarian cancer treatment.

### 6.3. Anticancer Activity Against Liver Cancer

Hepatocellular carcinoma is the predominant form of primary liver cancer and the sixth most frequently diagnosed malignancy worldwide. Despite this, therapeutic options for liver cancer remain limited, with nearly one million new cases and over 800,000 deaths reported annually [104,105]. Primary liver cancers encompass several pathological subtypes, including hepatocellular carcinoma, cholangiocarcinoma, and mixed hepatocellular-cholangiocarcinoma, with hepatocellular carcinoma being the most dominant, accounting for approximately 75–85% of cases globally. The incidence of hepatocellular carcinoma is notably high among patients with chronic liver diseases, such as hepatic fibrosis and cirrhosis associated with hepatitis B or C viral infection, metabolic liver disorders, alcohol abuse, particularly nonalcoholic fatty liver disease, and prolonged exposure to dietary carcinogens like aflatoxins and aristolochic acid. In China, the proportion of hepatocellular carcinoma among primary liver cancers is even higher, reaching nearly 93% [106,107,108].

Recent studies have further clarified the antitumor effects and underlying mechanisms of PEO against hepatocellular carcinoma through both *in vitro* and *in vivo* investigations. PEO showed significant inhibitory effects on hepatocellular carcinoma cell proliferation while exhibiting substantially lower cytotoxicity toward normal cells, particularly normal hepatocytes, indicating a favorable selectivity index. Mechanistically, PEO induced G_0_/G_1_ phase cell cycle arrest via both p53-dependent and -independent pathways involving key cell cycle-associated proteins. Concurrently, PEO activated the FAS-FASL-CASP8 signaling cascade, initiating extrinsic apoptotic pathways, while also increasing the BAX/BCL-2 ratio and promoting mitochondrial-mediated intrinsic apoptosis. These pro-apoptotic effects were closely linked to excessive intracellular reactive oxygen species (ROS) generation induced by PEO [92].

Notably, PEO demonstrated synergistic anticancer effects when combined with sorafenib, resulting in enhanced inhibition of hepatocellular carcinoma cell proliferation by modulating the Akt/mTOR signaling pathway and significant suppression of xenograft tumor growth. In animal models, PEO significantly reduced tumor burden, prolonged survival, and downregulated angiogenic signaling by suppressing the vascular endothelial growth factor (VEGF)/vascular endothelial growth factor receptor (VEGFR) axis, while inducing apoptosis in tumor tissues *in vivo*. Importantly, no significant systemic, physiological, or pathological toxicity was observed in treated animals. Collectively, these findings demonstrate that PEO (0–200 µg/mL) effectively suppresses hepatocellular carcinoma progression by inducing cell cycle arrest and activating multiple apoptotic pathways *in vivo* and *in vitro*. The low toxicity profile of PEO toward normal tissues further suggests a reduced risk of adverse effects, highlighting its potential for development as a therapeutic agent or functional food for the chemoprevention of hepatocellular carcinoma [92].

Beyond the essential oil constituents, individual phytochemicals isolated from patchouli significantly enhance its anti-liver-cancer activity. Pogostone, a major bioactive compound, has demonstrated concentration- and time-dependent cytotoxicity against liver cancer cell lines. Cytotoxicity assessments using the MTT assay, lactate dehydrogenase (LDH) release assay, and trypan blue exclusion test have shown notable reductions in cell viability and membrane integrity following pogostone treatment. Apoptotic induction was confirmed by diphenylamine assays, Annexin V-FITC staining, and real-time PCR. Pogostone significantly downregulated the expression of the anti-apoptotic gene *BCL-2* while markedly upregulating pro-apoptotic markers, including BAX, p53, and CASP3, thereby promoting apoptosis primarily by modulating the BAX/BCL-2 ratio [93].

Moreover, flavonoids derived from patchouli, such as pachypodol, along with other natural antioxidants and phytochemicals, have recently been suggested as complementary anticancer agents due to their antiproliferative and pro-apoptotic properties [109,110]. Notably, pachypodol has shown significant cytotoxicity against HepG2 liver cancer cells, with an IC_50_ of 0.55 mg/mL, highlighting its potential as a promising candidate for further research in liver cancer therapeutics [111].

These findings collectively demonstrate that constituents of patchouli, including PEO, pogostone, and flavonoids such as pachypodol, exhibit significant anticancer effects against hepatocellular carcinoma. They achieve this by coordinating cell-cycle arrest, activating both extrinsic and intrinsic apoptotic pathways, modulating oxidative stress, and suppressing angiogenic signaling. This highlights their strong potential as promising plant-derived agents for the prevention and therapeutic intervention of liver cancer.

### 6.4. Anticancer Activity Against Gallbladder Cancer

Gallbladder cancer is a highly aggressive malignancy of the biliary tract with pronounced geographic variation, exhibiting particularly great extent in India, South America, and East Asia [112,113,114]. Global epidemiological data suggest an annual incidence of approximately 1–2 cases per 100,000 individuals, with a higher prevalence observed in women than in men [115]. Owing to its asymptomatic early course, gallbladder cancer is frequently diagnosed at advanced stages, resulting in poor clinical outcomes. Moreover, standard therapeutic modalities, including chemotherapy and radiotherapy, provide limited efficacy, posing substantial challenges to improving patient prognosis [116].

Recent experimental evidence demonstrates that pogostone exerts notable anticancer activity against gallbladder carcinoma. In the human gallbladder cancer cell line SGC-996, pogostone markedly inhibited cell proliferation and colony formation. These effects were mechanistically associated with activation of apoptosis-related pathways, as indicated by increased expression of *CASP9*, *CASP3*, and poly (ADP-ribose) polymerase-1 (*PARP-1*), together with an elevated *BAX/BCL-2* ratio. In parallel, pogostone significantly suppressed cell-cycle progression by downregulating cyclins A, B, and D1 [91].

Collectively, these findings indicate that pogostone effectively inhibits gallbladder cancer cell growth by concurrently inducing apoptosis and arresting cell-cycle progression. This underscores its potential as a promising plant-derived anticancer agent for gallbladder cancer therapy.

### 6.5. Anticancer Activity Against Colorectal Cancer

Colorectal cancer is the third most commonly diagnosed malignancy and the second leading cause of cancer-related mortality worldwide [117]. Its occurrence is closely linked to Western lifestyle factors, such as excessive alcohol consumption, obesity, and a high intake of red and processed meats [118]. Individuals with chronic inflammatory bowel diseases, such as ulcerative colitis and Crohn’s disease, face a significantly heightened risk of colorectal cancer and thus require vigilant clinical monitoring [119]. Approximately 5% of colorectal cancer cases stem from hereditary syndromes associated with disease development, such as hereditary non-polyposis colorectal cancer (HNPCC) and familial adenomatous polyposis (FAP), while an additional ~20% of cases are linked to familial clustering. The majority of colorectal cancer cases (~75%) occur sporadically [118].

Patchouli has demonstrated significant anticancer activity against colorectal cancer in both *in vivo* and *in vitro* models. Treatment with patchouli extracts (0–80 μg/mL) significantly induced apoptosis and suppressed colorectal cancer cell proliferation, as evidenced by MTT-based assays. These effects were associated with G_0_/G_1_ cell-cycle arrest and modulation of apoptosis-related proteins. Notably, co-treatment with patchouli and 5-fluorouracil produced synergistic antiproliferative effects. *In vivo* studies further confirmed that patchouli inhibited tumor growth by inducing apoptosis without causing significant systemic toxicity, indicating a favorable safety profile [72].

Patchoulol has been identified as a principal contributor to patchouli’s anticancer effects in colorectal cancer. In HCT116 and SW480 cells, patchoulol inhibited proliferation and induced apoptosis in a dose-dependent manner by upregulating p21 and downregulating cyclin D1 and CDK4, consistent with cell-cycle arrest. Additionally, patchoulol suppressed histone deacetylase 2 activity and reduced *c-Myc* expression, while enhancing nuclear factor kappa B (NF-κB) transcriptional activity through p65 nuclear translocation, indicating coordinated regulation of cell-cycle control, epigenetic modulation, and apoptotic signaling [87].

Further studies have corroborated these findings in additional colorectal cancer models. In HT-29, LoVo, and Caco-2 cells, patchoulol (100 μM) markedly inhibited proliferation and induced G_1_-phase arrest, accompanied by suppression of β-catenin-dependent transcriptional activity. Consistently, oral administration of patchoulol (25–50 mg/kg) significantly reduced tumor number and burden *in vivo* in a dose-dependent manner [120]. Similar chemopreventive effects were observed in inflammation-associated colorectal tumorigenesis models, where patchoulol significantly reduced tumor formation in Apc^Min/+^ mice treated with dextran sulfate sodium, and showed antiproliferative and cell-cycle-arresting effects in Caco-2 cells [88].

Recent evidence has revealed olfactory receptor-mediated signaling as a novel mechanism underlying the anticancer activity of *P. cablin*. In an azoxymethane/dextran sulfate sodium-induced colorectal cancer mouse model and in CT26 and HCT116 colorectal cancer cells, patchouli oil significantly suppressed tumor progression by upregulating the ectopically expressed olfactory receptor OR13G1, activating the CaMKK2/AMPK signaling pathway, and inducing ferroptosis. Among the constituents of patchouli oil, patchouli alcohol (PA) was identified as the principal bioactive compound responsible for these antitumor effects. These findings identify the OR13G1–CaMKK2–AMPK axis and ferroptotic cell death as previously unrecognized mechanisms contributing to patchouli’s colorectal anticancer activity and further support PA as a promising natural therapeutic candidate for colorectal cancer [121].

In addition to essential oil constituents, patchouli leaf-derived flavonoids further contribute to its anticancer potential. Pachypodol exhibited antiproliferative activity against human Caco-2 colon cancer cells, with moderate cytotoxicity in brine shrimp lethality assays and measurable *in vitro* growth inhibition, suggesting a modest but significant anticancer effect [89].

These findings collectively demonstrate that bioactive constituents derived from patchouli, particularly crude extracts, patchoulol, and flavonoids like pachypodol, effectively inhibit the progression of colorectal cancer. They achieve this by integrating regulation of cell cycle arrest, apoptotic signaling, epigenetic modulation, inflammatory responses, and oncogenic signaling pathways, highlighting their strong potential as therapeutic and chemopreventive agents for colorectal cancer.

### 6.6. Anticancer Activity Against Lung Cancer

Lung cancer remains the most prevalent and deadly malignancy worldwide. It is broadly classified by cellular origin into small-cell lung cancer (SCLC) and non-small-cell lung cancer (NSCLC), with NSCLC accounting for the majority of cases [122,123,124]. Major histological subtypes include squamous cell carcinoma, adenocarcinoma, and neuroendocrine tumors such as large-cell neuroendocrine carcinoma, carcinoid tumors, and SCLC [122,125].

Patchoulol has demonstrated pronounced antitumor activity against NSCLC, particularly in A549 cell models, under both *in vivo* and *in vitro* conditions. It significantly inhibited tumor cell proliferation by inducing G_1_/S cell cycle arrest and stimulating mitochondrial-dependent apoptotic signaling, as evidenced by activation of CASP9 and CASP3. These effects were confirmed through multiple assays, including cell viability analysis, Hoechst 33,342 staining, TUNEL assays, and xenograft tumor suppression. Mechanistically, patchoulol exerted its anticancer effects by inhibiting the EGFR-ERK/MAPK signaling pathway, a central regulator of NSCLC proliferation. Reversal of these effects by exogenous epidermal growth factor further confirmed the dependence of patchoulol-induced apoptosis on EGFR pathway inhibition. Importantly, patchoulol displayed selective cytotoxicity toward cancer cells, with minimal toxicity observed in normal human cell lines, indicating a favorable safety profile [3].

In addition to apoptosis, patchoulol has been shown to induce autophagy-associated cell death in A549 cells. Treatment resulted in suppressed proliferation, accumulation of autophagic vesicles, and elevated LC3-II/LC3-I ratios, accompanied by reduced p62 expression, collectively indicating activation of autophagic pathways. However, the upstream molecular mechanisms governing patchoulol-induced autophagy remain unclear and warrant further investigation [126].

Patchoulol has also exhibited potent activity against drug-resistant NSCLC phenotypes. In both parental A549 and vincristine-resistant A549/V16 cells, patchoulol inhibited proliferation and colony formation while inducing G_0_/G_1_-phase arrest and apoptosis. These effects were mediated by ROS-induced DNA damage, activation of checkpoint kinases CHK1 and CHK2, and regulation of the p53/p21 and CDK2/cyclin E1 pathways. Apoptosis was primarily driven by intrinsic mitochondrial signaling, as evidenced by BAX upregulation and CASP activation. Notably, patchoulol reduced multidrug resistance and cancer stem cell-associated traits by downregulating P-glycoprotein (P-gp) and stemness markers such as CD44 and CD133. Moreover, combination treatment with patchoulol and cisplatin produced synergistic anticancer effects [94].

Collectively, these findings demonstrate that patchoulol exerts multifaceted anticancer effects against NSCLC by inducing cell-cycle arrest, mitochondrial apoptosis, and autophagy, while concurrently suppressing EGFR-driven oncogenic signaling, multidrug resistance, and cancer stem cell-associated phenotypes. These mechanisms underscore its strong therapeutic potential for both chemosensitive and drug-resistant lung cancer.

### 6.7. Anticancer Activity Against Nasopharyngeal Cancer

Nasopharyngeal carcinoma is a malignancy originating from the nasopharyngeal epithelium [127,128]. In the advanced stages of nasopharyngeal carcinoma, resistance to conventional chemotherapy and radiotherapy often develops, significantly limiting therapeutic efficacy. This diminished treatment responsiveness is mainly attributed to impaired apoptotic signaling in tumor cells, particularly due to the overexpression of the anti-apoptotic protein BCL-2. As a result, BCL-2 has become a crucial therapeutic target for restoring apoptosis and overcoming treatment resistance in nasopharyngeal carcinoma [95].

To discover potential natural inhibitors of BCL-2, bioactive compounds from patchouli were sourced from the KNApSAcK database and systematically assessed through molecular docking, followed by molecular dynamics simulations. Computational analyses pinpointed apigenin 7-(6″-p-coumarylglucoside), apigenin, and luteolin as promising candidates with significant inhibitory potential against BCL-2, based on their binding affinities and interaction profiles. Among these, apigenin 7-(6″-p-coumarylglucoside) showed the strongest predicted binding affinity (−9.9 kcal/mol), while luteolin and apigenin each exhibited binding energies of −7.2 kcal/mol. All three flavonoids were predicted to bind within the inhibitory pocket of BCL-2, primarily stabilized by hydrophobic interactions and van der Waals forces [95].

However, subsequent molecular dynamics simulations indicated that apigenin 7-(6″-p-coumarylglucoside) exhibited unstable binding over time, suggesting limited conformational persistence under dynamic physiological conditions. In contrast, luteolin and apigenin demonstrated more stable interaction profiles with BCL-2, suggesting a greater potential as functionally relevant inhibitors [95].

These findings collectively indicate that flavonoids derived from patchouli hold significant promise as alternative or complementary therapeutic agents for addressing radiotherapy- and chemotherapy-resistant nasopharyngeal carcinoma. Notably, luteolin and apigenin stand out as promising candidates for modulating apoptosis-related signaling by inhibiting *BCL-2*, thus meriting further experimental validation and translational exploration in nasopharyngeal carcinoma treatment strategies.

### 6.8. Anticancer Activity Against Skin Cancer

Skin cancer represents a major global health concern, with hundreds of thousands of new melanoma cases and more than 1.5 million non-melanoma cases reported annually worldwide [129,130]. Although melanoma occurs less frequently than non-melanoma skin cancers, it accounts for a disproportionately high mortality rate due to its aggressive behavior, strong metastatic capacity, and rapid dissemination to vital organs [131].

Patchoulol has demonstrated marked antitumor activity in both *in vitro* and *in vivo* melanoma models. In murine B16F10 melanoma cells, patchoulol selectively suppressed cell proliferation in a dose-(0–100 μM) and time-dependent manner, while inducing G_0_/G_1_ phase cell cycle arrest, and triggered apoptosis, as evidenced by chromatin condensation, DNA fragmentation, and apoptotic body formation. These effects were mechanistically associated with downregulation of *cyclin D1* and *CDK4*, driven by activation of both intrinsic and extrinsic apoptotic pathways [98].

Beyond its antiproliferative effects, patchoulol significantly suppressed melanoma cell migration and invasiveness by modulating epithelial–mesenchymal transition-related markers, including upregulation of E-cadherin and downregulation of vimentin, phosphorylated SMAD2/3, and matrix metalloproteinases MMP-2/9. In animal models, patchoulol markedly reduced tumor growth and, when combined with cisplatin, produced synergistic inhibition of colony formation and cell migration while attenuating chemoresistance [98].

Collectively, these findings identify patchoulol as a promising natural anticancer agent against melanoma, exerting multitarget effects through coordinated regulation of cell-cycle control, apoptotic signaling, metastatic pathways, and drug resistance.

### 6.9. Anticancer Activity Against Prostate Cancer

Prostate cancer is the most commonly diagnosed malignancy in men and a leading cause of cancer-related mortality, with more than 1.1 million new cases reported annually worldwide [132]. Owing to its prolonged clinical course and marked heterogeneity, prostate cancer is classified according to tumor localization, metastatic status, and androgen sensitivity, encompassing localized prostate cancer, metastatic hormone-sensitive prostate cancer, and metastatic castration-resistant prostate cancer [133,134,135].

Castration-resistant prostate cancer (CRPC) represents an advanced and aggressive disease stage that is unresponsive to androgen-deprivation and conventional hormonal therapies. Patchoulol has demonstrated significant anticancer activity against CRPC in both *in vivo* and *in vitro* models at concentrations of 0–100 μg/mL. In human CRPC cell lines (DU145 and PC-3), patchoulol markedly suppressed cell proliferation, migration, and invasion in a dose-dependent manner and strongly induced apoptosis. These effects were associated with mitochondrial membrane depolarization, increased levels of cleaved CASP3 and PARP, upregulation of the pro-apoptotic protein BAX, accompanied by downregulation of anti-apoptotic proteins and proliferative markers, including BCL-2, Ki67, and MCL-1 [97].

Mechanistic analyses revealed that patchoulol enhanced expression of the NF-κB inhibitor IκBα while reducing NF-κB p65 transcriptional activity. Inhibition of NF-κB signaling was accompanied by decreased expression of metastasis- and angiogenesis-related factors, including *MMP-2*, *MMP-7*, *MMP-9*, and *VEGF*. Notably, patchoulol disrupted NF-κB p65 binding to the MCL-1 promoter, thereby suppressing MCL-1 transcription. Functional studies confirmed that *p65* silencing potentiated patchoulol-induced apoptosis, whereas *MCL-1* overexpression attenuated cell death. Consistent with these findings, patchoulol significantly inhibited tumor growth in mouse xenograft models [97].

Collectively, these results demonstrate that patchoulol exerts potent anticancer effects against CRPC by suppressing NF-κB-mediated survival signaling, inhibiting metastatic and angiogenic pathways, and activating mitochondria-dependent apoptotic mechanisms, highlighting its promise as a therapeutic candidate for advanced prostate cancer.

### 6.10. Anticancer Activity Against Acute Myeloid Leukemia

Leukemia encompasses a diverse group of hematological malignancies marked by the unchecked proliferation of abnormal blood cells, typically originating in the bone marrow [136]. It continues to rank among the most prevalent cancers worldwide, placing a substantial burden on global health, especially in developing countries, due to premature mortality, while clinical outcomes are comparatively better managed in developed regions [137,138]. Among the various leukemia subtypes, acute myeloid leukemia is a highly aggressive malignancy arising from the myeloid lineage, associated with poor prognosis and limited therapeutic options [139].

The anticancer potential of patchouli extract and its underlying mechanisms have been explored in HL-60 human acute myeloid leukemia cells. Results from the MTT assay showed that patchouli extract, at concentrations ranging from 0 to 200 μg/mL, significantly inhibited HL-60 cell proliferation in a dose-dependent manner. This was accompanied by notable morphological changes, such as membrane blebbing and cell shrinkage. Cell cycle analysis indicated that the treatment induced G_0_/G_1_-phase arrest, while apoptosis was triggered in both dose- and time-dependent manners, as evidenced by increased DNA fragmentation and apoptotic body formation. Mechanistically, patchouli extract promoted G_0_/G_1_-phase accumulation by enhancing phosphorylation of the retinoblastoma protein (p-Rb), upregulating the cyclin-dependent kinase (CDK) inhibitor p21, and downregulating key cell cycle-regulatory proteins. Concurrently, the extract activated both extrinsic and intrinsic apoptotic pathways, ultimately leading to leukemic cell death [85].

Beyond whole extracts, the primary sesquiterpene component, patchoulol, has shown significant antiproliferative and pro-apoptotic effects in human leukemia MV4-11 cells. Treatment with patchoulol significantly reduced cell proliferation, with MV4-11 cells showing the greatest sensitivity among the tested cancer cell lines, including HCT116, HepG2, A549, A375, 4T1, and THP-1, as well as human embryonic kidney 293A cells. Apoptotic indicators, such as nuclear shrinkage and chromatin condensation, were evident following patchoulol exposure, and flow cytometric analysis confirmed a dose-dependent increase in apoptotic cell populations. Mechanistic studies further revealed that patchoulol-induced apoptosis was associated with downregulation of NF-κB signaling and phosphorylated pyruvate kinase M2 (p-PKM2), along with increased CASP3 protein expression, indicating activation of a caspase-dependent apoptotic pathway [86].

**Figure 3:**
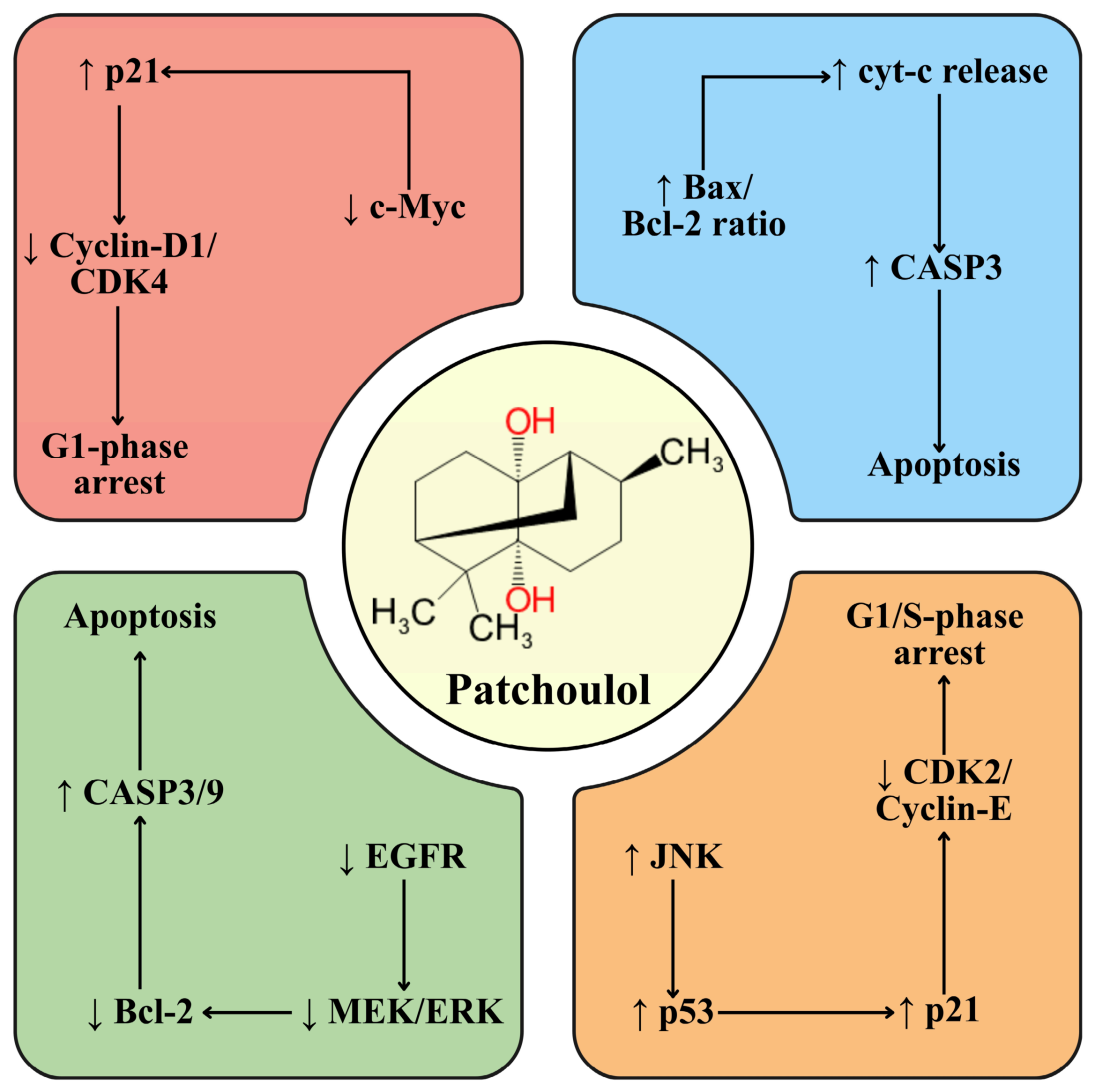
Proposed molecular mechanisms underlying the anticancer activity of patchoulol. This schematic illustrates the primary signaling pathways that patchoulol modulates in cancer cells. Patchoulol induces cell cycle arrest at the G_1_ and G_1_/S phases by downregulating *c-MYC*, cyclin D1/CDK4, and CDK2/cyclin E, while simultaneously upregulating the cell cycle inhibitor p21, partially mediated through JNK-p53 signaling. At the same time, patchoulol promotes apoptosis by activating intrinsic mitochondrial pathways, as indicated by elevated BAX/BCL-2 ratios, mitochondrial cytochrome c release, and activation of CASP9 and CASP3. Additionally, patchoulol inhibits EGFR-MEK/ERK signaling, reducing *BCL-2* expression, and further enhancing caspase-dependent apoptotic responses. Together, these coordinated effects on cell cycle regulation and apoptotic signaling pathways contribute to patchoulol’s broad-spectrum anticancer activity.

These findings collectively demonstrate that extracts and phytochemicals derived from patchouli leaves exhibit broad-spectrum anticancer activity against a wide range of malignancies, including endometrial, ovarian, liver, gallbladder, colorectal, lung, nasopharyngeal, skin, and prostate cancers, as well as acute myeloid leukemia. Despite differences among cancer types, patchouli-derived bioactive compounds consistently exert their anticancer effects through several conserved molecular mechanisms, including induction of apoptosis, cell-cycle arrest, suppression of proliferative and survival signaling pathways (e.g., PI3K/Akt, MAPK, and NF-κB), modulation of oxidative stress, and inhibition of tumor growth and metastasis. Among these constituents, patchoulol is the most extensively investigated compound, with consistent evidence from both *in vitro* and *in vivo* studies demonstrating its ability to regulate cell-cycle progression and activate both intrinsic and extrinsic apoptotic pathways. As summarized in Figure 3, patchoulol modulates key oncogenic signaling networks involving cell-cycle regulators, mitochondrial apoptosis, and growth factor-mediated pathways, providing a unified mechanistic framework for the anticancer activities of patchouli-derived phytochemicals. Collectively, these findings identify patchoulol as a representative lead compound and highlight the promise of patchouli-derived bioactive constituents as multi-target anticancer agents worthy of further mechanistic, pharmacological, and translational investigation.

## 7. Challenges and Limitations

Despite the growing interest in the anticancer potential of patchouli leaves, several challenges continue to hinder their translational development. A major constraint is the pronounced phytochemical variability associated with geographical origin, climate, soil conditions, cultivation practices, harvesting stage, and genetic diversity among cultivars. This variability may lead to inconsistent profiles and concentrations of bioactive compounds, resulting in differences in pharmacological activity and therapeutic efficacy [17]. Such heterogeneity complicates reproducibility, quality control, and standardization, while the molecular mechanisms underlying several reported pharmacological effects remain insufficiently elucidated [59]. Moreover, robust and large-scale clinical trials validating the therapeutic potential of patchouli-derived products remain scarce [140].

Methodological limitations further restrict progress. The absence of standardized extraction and processing protocols can lead to substantial variations in phytochemical yield, purity, and quality, as extraction techniques and distillation parameters strongly influence the composition of patchouli-derived preparations [141]. The high sesquiterpene content of patchouli leaves may also require prolonged distillation, reducing processing efficiency and emphasizing the need for optimized or innovative extraction strategies [38]. In addition, weaknesses in experimental design, including small sample sizes, inadequate controls, insufficient biological replication, and reliance on conventional analytical approaches, may compromise data reliability and overlook minor but biologically relevant constituents [23]. Product quality is another important concern, because contamination with heavy metals, pesticide residues, microorganisms, or adulterants may pose safety risks and limit the scalability and sustainability of patchouli-derived medicinal products [38].

Regulatory and safety considerations represent additional barriers. The absence of harmonized quality-control standards and the variability of regulatory frameworks governing herbal products contribute to inconsistencies in product efficacy, safety, and market acceptance [142]. Regulatory approval requires extensive pharmacological, toxicological, and clinical evaluation, making commercialization costly and time-consuming [143]. Comprehensive safety assessments addressing potential adverse effects, long-term toxicity, dose-dependent responses, and interactions with conventional drugs are therefore essential for ensuring regulatory compliance and establishing confidence in patchouli-derived therapeutic products [144].

Limited bioavailability is also a significant challenge affecting the therapeutic use of patchouli phytochemicals. Poor aqueous solubility, incomplete gastrointestinal absorption, rapid metabolism, and differences in compound composition among plant parts and developmental stages may substantially influence systemic exposure and biological efficacy [145]. Extraction methods, formulation strategies, dosing conditions, and interindividual variability may further affect clinical outcomes, while determining effective and safe dosing regimens requires extensive pharmacological validation [20]. Strategies that improve solubility, stability, absorption, and pharmacokinetic consistency are therefore essential for maximizing the therapeutic potential of patchouli-derived compounds [146].

Although the anticancer potential of PA has been widely demonstrated in preclinical studies, its clinical translation remains constrained by limited pharmacokinetic, formulation, and long-term safety data. One of the major pharmaceutical challenges is PA’s poor aqueous solubility, which limits its oral bioavailability. Several formulation strategies have consequently been investigated to improve its solubility, dissolution, stability, and systemic exposure. Solid dispersions formulated with Eudragit achieved prolonged supersaturation and maintained higher dissolved PA concentrations, thereby improving its dissolution profile [147]. Similarly, poloxamer-based solid dispersion systems containing Poloxamers 188 and 407 enhanced PA solubility and dissolution while offering a relatively simple, rapid, cost-effective, and potentially scalable preparation process [148]. β-Cyclodextrin inclusion complexes have also been developed to improve PA stability and dissolution characteristics, as demonstrated using differential scanning calorimetry (DSC), Fourier-transform infrared spectroscopy (FT-IR), powder X-ray diffraction (PXRD), and scanning electron microscopy (SEM) [149].

Available pharmacokinetic evidence indicates that PA follows a two-compartment open model with linear elimination kinetics in experimental animals. Following intravenous administration in rats, PA displayed relatively rapid systemic disposition, whereas oral administration resulted in measurable plasma exposure, with parameters such as the time to maximum plasma concentration (Cmax), area under the concentration–time curve (AUC), Time to reach Cmax (Tmax), and elimination half-life characterized using gas chromatographic and gas chromatography–mass spectrometry (GC-MS) methods [150,151]. Preliminary metabolism studies have additionally identified hydroxylated metabolites of PA in rabbit liver, providing initial evidence concerning its metabolic fate [152]. However, these investigations were conducted predominantly in healthy or non-cancer animal models. Consequently, the effects of tumors, altered cancer metabolism, anticancer co-medications, and disease-associated physiological changes on PA absorption, distribution, metabolism, and elimination remain largely unknown.

Available toxicological studies suggest that PA has relatively low acute toxicity in experimental animals. High maximum tolerated doses following oral administration and reported median lethal dose (LD_50_) values indicate a comparatively favorable short-term safety profile. Nevertheless, the available evidence is mainly limited to acute or short-duration toxicity assessments, and poor PA solubility has complicated the determination of precise toxicity thresholds in some studies. Comprehensive evaluations of repeated-dose and chronic toxicity, reproductive and developmental toxicity, genotoxicity, immunotoxicity, carcinogenicity, allergic reactions, local irritation, organ-specific toxicity, and potential herb–drug interactions remain insufficient [153]. These limitations prevent definitive conclusions regarding the long-term safety of PA and other patchouli-derived bioactive constituents.

Importantly, the currently available formulation, pharmacokinetic, metabolism, and toxicity data were generated mainly in non-cancer settings and therefore cannot be directly extrapolated to anticancer treatment. Cancer-specific information concerning plasma half-life, tumor accumulation, biodistribution, pharmacologically active concentrations, therapeutic windows, optimal dosing regimens, and interactions with chemotherapy remains scarce. Moreover, existing delivery approaches have primarily focused on improving solubility and oral absorption rather than achieving tumor-selective accumulation or controlled release. The development of targeted delivery systems, including nanoparticle-, liposome-, polymer-, or ligand-based formulations, may improve the stability, bioavailability, tumor targeting, and therapeutic index of PA; however, their efficacy and safety require systematic validation in relevant cancer models [12].

Overall, the therapeutic development of patchouli leaves remains limited by phytochemical variability, non-standardized methodologies, regulatory challenges, inconsistent product quality, poor bioavailability, insufficient cancer-specific pharmacokinetic evidence, and incomplete long-term safety evaluation. Addressing these barriers will require standardized cultivation and extraction procedures, rigorous chemical characterization, well-designed pharmacological studies, cancer-specific biodistribution and pharmacokinetic investigations, advanced targeted formulation strategies, and comprehensive repeated-dose and long-term toxicological assessments. Interdisciplinary collaboration among phytochemists, pharmacologists, formulation scientists, toxicologists, oncologists, and regulatory authorities will be essential for facilitating the successful preclinical and clinical development of patchouli-derived anticancer agents.

## 8. Future Perspectives

Future research on patchouli leaves is expected to be driven by advances in analytical, biotechnological, and translational approaches to realize their anticancer potential fully. Developments in high-resolution analytical platforms, such as MS and HPLC, have enabled more comprehensive characterization of phytochemical profiles and will continue to improve understanding of the bioactive constituents responsible for therapeutic effects [39]. Simultaneously, the increasing application of omics technologies, including proteomics, metabolomics, transcriptomics, and genomics, provides powerful tools for elucidating the biosynthetic pathways and regulatory networks governing the production of therapeutically important compounds [154]. When integrated with genetic and metabolic engineering strategies, these approaches may enable the targeted enhancement of key metabolites and support the sustainable production of patchouli-derived bioactive compounds for pharmaceutical and industrial applications [155].

From a therapeutic perspective, patchouli represents a promising but underexplored source of anticancer agents [156]. Although numerous bioactive compounds have been identified, systematic isolation, validation, and structure–activity relationship analyses of the principal anticancer constituents remain limited. This highlights the need for bioactivity-guided fractionation, compound standardization, and integrative phytochemical investigations to identify the compounds primarily responsible for the observed anticancer effects [157,158]. Furthermore, while antiproliferative, pro-apoptotic, and antimetastatic activities have been reported in experimental models, the precise molecular mechanisms, particularly those involving oncogenic signaling pathways, apoptosis, cell-cycle regulation, oxidative stress, ferroptosis, autophagy, and modulation of the tumor microenvironment, require further validation using well-characterized cellular and animal models [12]. The potential synergistic or chemosensitizing interactions between patchouli-derived compounds and conventional anticancer drugs should also be systematically evaluated to determine whether these compounds can enhance therapeutic efficacy, reduce drug resistance, or permit lower chemotherapy doses.

To facilitate clinical translation, future studies should prioritize cancer-specific pharmacokinetic and pharmacodynamic investigations together with the development of advanced targeted delivery systems and comprehensive long-term safety evaluations. Particular emphasis should be placed on characterizing the absorption, distribution, metabolism, elimination, tumor biodistribution, and therapeutic window of major bioactive compounds under clinically relevant conditions. The integration of pharmacokinetic and pharmacodynamic analyses with efficacy and safety assessments will be essential for optimizing therapeutic dosing, enhancing bioavailability and tumor targeting, minimizing off-target toxicity, and accelerating the successful translation of promising preclinical findings into well-designed clinical trials and ultimately clinical application.

Progress in this field will require coordinated efforts across multiple research areas. Comprehensive phytochemical profiling of different plant parts, cultivars, geographical origins, and developmental stages will be essential for capturing chemical diversity and identifying optimal sources of bioactive compounds [58]. Standardized cultivation, harvesting, extraction, processing, and quality-control procedures should be established to ensure reproducibility across studies. Expanding mechanistic investigations will be necessary to validate pharmacological targets and signaling pathways [159], while carefully designed preclinical studies should precede well-controlled clinical trials evaluating efficacy, safety, pharmacokinetics, tolerability, and potential interactions in humans [160].

Ultimately, interdisciplinary collaboration among phytochemists, pharmacologists, oncologists, toxicologists, formulation scientists, and bioinformaticians will accelerate the translation of patchouli leaf-derived compounds into clinically useful anticancer therapeutics and facilitate their development as novel anticancer drugs or effective adjuvant therapies [57,154].

## 9. Conclusions

This review provides a comprehensive synthesis of the current evidence regarding the anticancer potential of *P. cablin*, highlighting its phytochemical composition and the molecular mechanisms reported in experimental cancer models. Patchouli leaves contain diverse volatile and non-volatile bioactive constituents, including sesquiterpenes, flavonoids, glycosides, phytosterols, phenolic acids, and organic acids. Among these, patchoulol, pogostone, pachypodol, and other phytochemicals have received the greatest attention because of their reported anticancer activities. Collectively, the available evidence demonstrates that patchouli-derived extracts and bioactive compounds exhibit antiproliferative, pro-apoptotic, cell cycle-regulating, and anti-metastatic activities across multiple cancer cell lines and several animal models.

Experimental studies suggest that these effects are mediated through modulation of multiple molecular pathways, including NF-κB, PI3K/AKT, AKT/mTOR, EGFR/ERK, oxidative stress, mitochondrial apoptosis, and epigenetic regulation. Several studies have also reported enhanced chemosensitivity and reduced drug resistance when patchouli-derived compounds were combined with conventional anticancer agents. However, these findings are derived predominantly from *in vitro* experiments and preclinical animal models and should therefore be interpreted with caution.

Despite these promising experimental observations, the clinical translation of patchouli-derived compounds remains at a very early stage. Evidence regarding pharmacokinetics, bioavailability, formulation strategies, long-term toxicity, drug interactions, and clinical efficacy is still limited. Moreover, phytochemical variability, the absence of standardized extraction procedures, and the lack of rigorous clinical trials remain major barriers to therapeutic development. Consequently, the current evidence is insufficient to support clinical application or therapeutic recommendations.

Overall, the available literature indicates that patchouli is a promising source of bioactive molecules for future anticancer drug discovery. Nevertheless, well-designed pharmacokinetic studies, comprehensive toxicological evaluations, standardized formulations, and carefully conducted clinical trials are essential before the therapeutic value of patchouli-derived compounds can be established. This review provides a foundation for future research by summarizing the current evidence and identifying the key knowledge gaps that should be addressed to facilitate the translational development of patchouli-derived anticancer agents.

## Figures and Tables

**Table 1:** Representative volatile phytochemical constituents identified in *P. cablin* (patchouli) leaves.

Compound Name	Chemical Class	Molecular Formula	Extraction Technique	Analytical Identification	References
(−)-camphor	Monoterpenes	C_10_H_16_O	Steam distillation	GC × GC–TOF-MS	[46]
β-pinene		C_10_H_16_			[46,47,48]
β-phellandrene		C_10_H_16_			[46]
α-elemenone	Sesquiterpenes	C_15_H_22_O			[46]
pogostol		C_15_H_26_O			[47]
(−)-α-selinene		C_15_H_24_		GC-MS	[48]
α-bulnesene		C_15_H_21_			[46,47]
α-humulene		C_15_H_24_			[47]
β-caryophyllene		C_15_H_24_			[46,47,48]
β-selinene		C_15_H_24_			[47,48]
*cis*-β-elemene		C_15_H_24_			[47]
cycloseychellene		C_15_H_24_			[47]
seychellene		C_15_H_24_			[47,49,50]
trans-caryophyllene		C_15_H_24_			[51]
zizanal		C_15_H_22_O			[47]
δ-elemene		C_15_H_24_			[47,52]
γ-gurjunene		C_15_H_24_			[48,53]
α-, β-, δ-guaiene		C_15_H_24_		GC-MS, GC	[47,54]
germacrene D		C_15_H_24_			[47,53]
limonene		C_10_H_16_			[47]
patchouli alcohol		C_15_H_26_O			[47]
δ-patchoulene		C_15_H_24_		GC-MS, GC, NMR	[47,54]
β-elemene		C_15_H_24_		GC-MS, NMR	[52]
α-, β-patchoulene		C_15_H_24_		GC-TOF-MS	[47,55]
globulol		C_15_H_26_O		GC-TOF-MS, GC	[56]
nortetrapatchoulol		C_14_H_24_O		GC-TOF-MS, GC-MS	[47]
norpatchuolenol	Alcohols	C_14_H_22_O		GC-MS	[47]
3-iso-thujopsanone	Ketones	C_15_H_24_O			[47]
pogostone		C_12_H_16_O_4_			[46,47]

**Table 2:** Representative non-volatile phytochemical constituents identified in *P. cablin* (patchouli) leaves.

Compound Name	Chemical Class	Molecular Formula	Extraction Technique	Analytical Identification	References
pachypodol	Flavonoids	C_16_H_12_O_6_	Ultrasonic-assisted 70% ethanol extraction	GC	[46,62]
3,5-dihydroxy-7,4′-dimethoxy-flavone		C_17_H_14_O_6_	Reflux extraction with 70% aqueous ethanol	HPLC-DAD	[48]
4′,5,7-trihydroxyflavone		C_15_H_10_O_5_			[48]
4′,5-dihydroxy-3,3′,7-trimethoxyflavone		C_18_H_18_O_7_			[48]
5-hydroxy-3,3′,4′,7-tetramethoxyflavone		C_18_H_18_O_6_			[48]
5-hydroxy-3,4′,7-trimethoxyflavone		C_17_H_16_O_6_			[48]
5-hydroxy-7,3′,4′-trimethoxyflavanone		C_17_H_16_O_6_			[48]
apigenin		C_15_H_10_O_5_	Ultrasonic-assisted 70% ethanol extraction		[46,62]
rhamnetin		C_16_H_12_O_7_			[62]
isocrenatoside	Glycosides	C_29_H_34_O_15_	Maceration with 50% aqueous ethanol	GC	[46,54]
7R-campeoside II		C_29_H_36_O_16_		HPLC	[63]
7S-campeoside II		C_29_H_36_O_16_			[63]
verbascoside		C_29_H_36_O_15_			[64]
2″,3″-O-acetylmartynoside		C_35_H_46_O_17_		HPLC-DAD	[63]
3′-methoxyisocrenatoside		C_30_H_34_O_16_			[63]
actinoside		C_36_H_44_O_20_			[57]
osmanthuside B		C_29_H_36_O_13_			[63]
pedicularioside G		C_17_H_26_O_11_			[64]
crenatosides		C_29_H_34_O_15_		TLC, HPLC	[65]
acteoside		C_29_H_36_O_15_		TLC, HPLC, HPLC-Q-TOF-MS	[65,66]
isoacteosides		C_29_H_36_0_15_			[65,67]
epifriedelinol	Triterpenoids	C_30_H_52_O	Reflux extraction with petroleum ether and chloroform	NMR, IR, MS, UV	[46]
friedelin		C_30_H_50_O			[46]
methyl oleanolate		C_31_H_50_O_3_			[46]
oleanolic acid		C_30_H_48_O_3_			[46]
cytosporone V	Other compounds	C_13_H_16_O_5_	Ultrasonic-assisted 70% ethanol extraction	HPLC	[46]
cytosporone W		C_12_H_15_O_5_			[63]
isolariketoester		C_33_H_39_O_10_			[63]
lariketoester		C_33_H_39_O_10_			[63]

**Table 3:** Anticancer activities of patchouli-derived extracts and phytochemicals across different cancer models.

Cancer Type	Compound	Experimental Model	Study Type	Molecular Target/ Signaling Pathway	Dosage & Duration	Reference
Acute myeloid leukemia	PCAE	HL-60 cells	*In vitro*	p21, p-Rb, cyclins; extrinsic and intrinsic apoptosis pathways (caspase activation)	0–200 µg/mL, 24–72 h	[85]
	Patchoulol	MV4-11 cells	*In vitro*	PKM2, NF-κB, caspase-3	25–100 µM, 24–48 h	[86]
Colorectal cancer	PCAE	HCT116 cells	*In vitro + in vivo*	G_0_/G_1_ cell-cycle arrest, apoptosis-related proteins; synergistic effect with 5-FU	10–80 µg/mL (*in vitro*) and 30 mg/kg (*in vivo*)	[72]
	Patchoulol	HT-29	*In vitro*	HDAC2, c-Myc, NF-κB, p21, cyclin D1, CDK4	50–100 µM, 24–48 h	[87]
		Caco-2 cells	*In vitro + in vivo*	G_1_-phase arrest, AMPK, Akt phosphorylation, glucose uptake	25–100 µM, 24 h	[88]
	Pachypodol	Caco-2 cells	*In vitro*	Cytotoxicity against CaCo-2 colon cancer cells (IC_50_ = 185.6 μM); no specific molecular target investigated	IC_50_ ≈ 185 µM, 48 h	[89]
Endometrial cancer	PCAE	Ishikawa cells	*In vitro*	Caspase-3, caspase-9, AIF-mediated apoptosis	0–4 mg/mL, 24–48 h	[90]
Gallbladder cancer	Pogostone	SGC-996 cells	*In vitro*	Mitochondrial apoptosis (Bax/Bcl-2, caspase-9/caspase-3, PARP), cyclin D1, cyclin A, cyclin B1, S-phase arrest	25–100 µg/mL, 24–48 h	[91]
Liver cancer	PEO	HepG2 cells	*In vitro + in vivo*	ROS-mediated DNA damage, p53, Fas/FasL/caspase-8, Bax/Bcl-2, Akt/mTOR, VEGF/VEGFR	0–200 µg/mL (*in vitro*) and 25–50 mg/kg (*in vivo*)	[92]
	Pogostone	HepG2 cells	*In vitro*	Bax/Bcl-2, p53, caspase-3	10–100 µg/mL, 24–48 h	[93]
Lung cancer	Patchoulol	A549 cells	*In vitro + in vivo*	EGFR/MAPK, mitochondrial apoptosis (caspase-9/caspase-3)	50–100 µg/mL and 25 mg/kg	[3]
		A549 cells, and NSCLC cells	*In vitro*	ROS–CHK1/CHK2, p53/p21, CDK2/cyclin E1, Bax/caspase-9/caspase-3, P-glycoprotein, CD44, CD133	150–300 µM, 24 h	[94]
Nasopharyngeal cancer	Apigenin	NPC cell lines	In silico (molecular docking & molecular dynamics)	BCL-2 inhibition (apigenin, rhamnetin, apigenin-7-(6″-p-coumarylglucoside))	20–80 µM	[95]
Ovarian cancer	Pogostone	OVCAR-3 cells	*In vitro*	PTEN, DACT1, Bax/Bcl-2, caspase-3, caspase-8, caspase-9, CCND1, CDK4	IC_50_ ≈ 90 µg/mL, 24–48 h	[96]
Prostate cancer	Patchoulol	DU145, and PC-3 cells	*In vitro + in vivo* (xenograft)	NF-κB/IκBα/p65, Mcl-1, Bax/Bcl-2, cleaved caspase-3, PARP, MMP-2, MMP-7, MMP-9, VEGF	25–100 µg/mL and 30 mg/kg	[97]
Skin cancer	Patchoulol	B16F10 cells	*In vitro + in vivo*	TGF-β/Smad2/3, E-cadherin, vimentin, MMP-2, MMP-9, G_0_/G_1_ cell-cycle arrest, apoptosis	0–100 µM and 20 mg/kg	[98]

## Data Availability

No new data were created or analyzed in this study. Data sharing is not applicable to this article.

## Abbreviations

The following abbreviations are used in this manuscript:

AIF	Apoptosis-inducing factor
CASP3/8/9	Caspase-3/-8/-9
CDK	Cyclin-dependent kinase
CRPC	Castration-resistant prostate cancer
DACT1	Dishevelled-binding antagonist of β-catenin 1
DNA	Deoxyribonucleic acid
EGFR	Epidermal growth factor receptor
FAP	Familial adenomatous polyposis
FIGO	International Federation of Gynecology and Obstetrics
GC	Gas chromatography
GC-MS	Gas chromatography–mass spectrometry
GEP	Gene expression profiling
HNPCC	Hereditary non-polyposis colorectal cancer
HPLC	High-performance liquid chromatography
HPLC-Q-TOF-MS	High-performance liquid chromatography–quadrupole time-of-flight mass spectrometry
HSCCC	High-speed counter-current chromatography
LDH	Lactate dehydrogenase
MCL-1	Myeloid cell leukemia-1
MMP	Matrix metalloproteinase
MS	Mass spectrometry
MTT	3-(4,5-Dimethylthiazol-2-yl)-2,5-diphenyltetrazolium bromide
NF-κB	Nuclear factor kappa B
NSCLC	Non-small-cell lung cancer
PARP-1	Poly(ADP-ribose) polymerase-1
PCAE	*Pogostemon cablin* aqueous extract
PCR	Polymerase chain reaction
PEO	Patchouli essential oil
PI3K/AKT	Phosphoinositide 3-kinase/Protein kinase B signaling pathway
PTEN	Phosphatase and tensin homolog
ROS	Reactive oxygen species
SCLC	Small-cell lung cancer
TLC	Thin-layer chromatography
TUNEL	Terminal deoxynucleotidyl transferase dUTP nick-end labeling
VEGF	Vascular endothelial growth factor
